# The Development and Characterization of Layered Pellets Containing a Combination of Amorphized Amlodipine Besylate and Hydrochlorothiazide Using a High-Shear Granulator

**DOI:** 10.3390/ph18101496

**Published:** 2025-10-05

**Authors:** Azza A. K. Mahmoud, Krisztina Ludasi, Dorina Gabriella Dobó, Dániel Sebők, Ákos Kukovecz, Viktória Hornok, Kadosa Sajdik, Tamás Szabó, Tamás Sovány, Géza Regdon, Katalin Kristó

**Affiliations:** 1 Institute of Pharmaceutical Technology and Regulatory Affairs, University of Szeged, Eötvös u. 6, H-6720 Szeged, Hungary; 2 Department of Applied and Environmental Chemistry, University of Szeged, Rerrich B. tér 1, H-6720 Szeged, Hungary; 3 Department of Physical Chemistry and Materials Science, University of Szeged, Rerrich B. tér 1, H-6720 Szeged, Hungary

**Keywords:** solventless granulation, layered pellets, high-shear granulator, partial amorphization

## Abstract

**Background/Objective:** The high-shear granulator is considered an effective piece of equipment for layering pelletization because it enhances drug amorphization and improves drug dissolution. This study aimed to apply a high-shear granulator to prepare layered pellets containing a combination of hydrochlorothiazide and amlodipine besylate with improved physicochemical properties. **Methods:** Different molar ratios (2:1, 1:1, and 1:2) of the hydrochlorothiazide and amlodipine besylate mixture were deposited on the surface of the inert spheres of the microcrystalline cellulose (MCC) core by the mechanical effect of the high impeller speed. The resulting layered pellets were characterized using X-ray powder diffractometry (XRPD) and differential scanning calorimetry (DSC) to estimate the degree of the drug amorphization, and consequently a dissolution test was performed to determine the degree of the enhancement of the percentage of release. Additionally, micro-computed tomography (micro-CT) and a texture analyzer were used to determine the morphological characteristics and hardness of the resulting pellets, and then a stability study was performed. **Results:** On the basis of the micro-CT images, the MCC core was successfully loaded with a uniform layer of the drug combination at the pellet surface, which exhibited higher diameters than pure cellets. Furthermore, the drug combination in layered pellets was partially amorphized with a lower crystallinity percentage, a lower intensity, a broadening of the hydrochlorothiazide melting peak, and a higher cumulative release of both drugs with good stability, except pellets with a molar ratio of 1:2 that were recrystallized with a higher crystallinity percentage of 79.9%. **Conclusions:** Modifying the physical form and dissolution behavior of the hydrochlorothiazide and amlodipine besylate combination was achieved by single-step layering pelletization.

## 1. Introduction

One of the most commonly used methods for improving the dissolution of poorly water-soluble drugs is the conversion of the crystalline form to an amorphous form carried out via a thermodynamic transformation by a melting-based or solvent-based method [1]. These methods have numerous drawbacks, including their high energy consumption, the large amount of polymer needed, the hygroscopicity of the polymer, lower stability, the risk of toxicity and drug degradation related to the residual solvent (solvent-based method), and the high temperature (melt-based process) [2,3]. On the other hand, applying layering pelletization has a beneficial effect on the drug distribution and consistent dose administration [4]. In this technique, drugs in the form of a solution or suspension or powder are loaded onto inert pellet cores, such as microcrystalline cellulose, tartaric acid, sucrose, and isomalt [5,6], that act as carriers, and it contributes to the masking of unpleasant tastes and improving the dissolution rate of drugs [7]. Among these pellet cores, microcrystalline cellulose is widely used in layering pelletization, as it has an ideal roundness with a smooth surface, lower friability, and higher mechanical resistance than sugar cores [8,9]. The layering pelletization of liquid drug requires the optimization of different parameters, such as the application rate, atomization type, and drying process, in addition to the type and concentration of the binder that can adversely affect the surface of the pellet, while the layering of the drug powder is associated with shorter process times and less process parameters due to the fact that it does not require a liquid binder, making it a good choice for moisture-sensitive drugs [9,10,11]. Additionally, applying mechanical force in the layering pelletization process using a fluidized bed or high-shear granulator in a single step is considered a highly stabilized and economically alternative technique without the utilization of solvents or heat [1,2,12]. Although this process can be performed through a single step and can change the physical properties of drugs (amorphization of crystalline drugs), it faces a huge risk with regard to recrystallization and the failure of the production of a single phase due to the excessive mechanical force and heat created within the granulator. This can be minimized by the appropriate selection of the process temperature [2] and the preparation of the co-amorphous form using a stabilizer of a low-molecular-weight co-former or another drug(s) with a high drug binding affinity that reduces molecular mobility by forming a constant chemical bond such as, a hydrogen bond, ionic bond, and π-π bond, in addition to using a stabilizer with a high Tg (glass transition temperature). This ensures an increase in the Tg of the co-amorphous material, producing more than the Tg of the required drug, and decreases the recrystallization probability [13,14,15]. Kondo et al. prepared amorphous indomethacin and loaded it on the surface of microcrystalline cellulose spheres using a high-shear granulator. Despite the higher solubility and dissolution rate of the resulting pellets, they had low stability, and this was overcome by adding L-arginine, which formed a co-amorphous system with indomethacin with enhanced dissolution and better stability than the crystalline drug form and the pellets containing the amorphous indomethacin alone [1,2]. Furthermore, drug–drug amorphization has many beneficial effects in the manufacture of different drug combinations, with improved solubility, harmonized synergetic pharmacological effects, fewer side effects, and therapeutic doses required, as long as it matches the ratio of the formulated drugs [16,17,18].

Amlodipine besylate is an antihypertensive agent that acts as a calcium channel blocker. It has a weak basic structure and a crystalline physical form with a melting point of 200 °C and a pKa of 9.1, which is classified in the biopharmaceutical classification system as BCS I [19], and it amorphizes when it is prepared as a solid dispersion with polyvinylpyrrolidone K30 at a weight ratio of 5:95, which improves its physical stability [20]. Furthermore, the co-amorphous system of amlodipine besylate with candesartan in the molar ratio 1:1 demonstrates better dissolution behavior than crystalline drugs, in addition to having better stability in the formulation that contained polyvinylpyrrolidone [21]. On the other hand, hydrochlorothiazide is an antihypertensive agent that acts as a diuretic thiazide and has been classified as BCS IV, which has poor solubility and permeability in addition to a crystalline structure with a melting point of 270 °C and a pKa of 9.09 [22,23]. Many researchers have mentioned improving solubility by using a solid dispersion with cyclodextrin [24], a water-soluble co-former (arginine) [25], a drug co-former like telmisartan [26] or atenolol [27], and simvastatin [28]. The combination of amlodipine besylate and hydrochlorothiazide is frequently used as a treatment for hypertension because it has a greater therapeutic effect in lowering blood pressure than single drugs in addition to minimizing metabolic side effects of hydrochlorothiazide, improving the blood’s level of low-density lipoprotein (LDL) and high-density lipoprotein (HDL). Moreover, the application of a high-shear granulator in the co-amorphization of the drug combination is considered a good technique that produces pellet dosage forms with improved physiochemical properties and therapeutic outcomes [29]. Many researchers have focused on preparing amorphous powders using traditional amorphization techniques, which require an additional manufacturing process to obtain the final dosage form. However, the conversion of the drug alone and with a co-former to an amorphous form by layering pelletization has been mentioned in only two studies [2,30]. The combined amorphization and layered pelletization process of drug combinations using a high-shear granulator has not been investigated and is presented for the first time in this study.

This study focused on the formulation of a multiparticle system loaded with an amlodipine besylate and hydrochlorothiazide combination using a novel approach of solvent-free direct pelletization in a high-shear granulator.

## 2. Results and Discussion

### 2.1. Differential Scanning Calorimetry (DSC)

**Figure 1:**
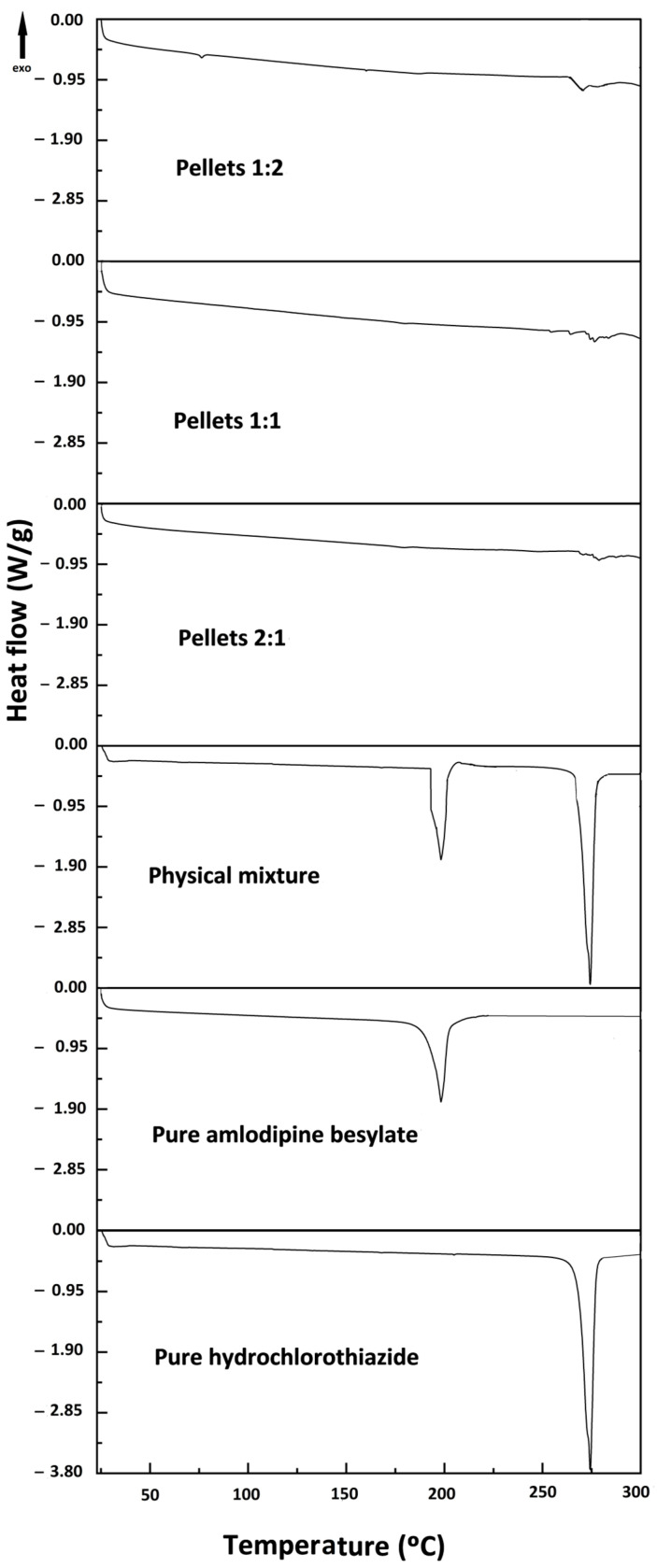
DSC thermograms of pure drugs and layered pellets containing different molar ratios.

The thermal behavior of the pure drugs and the resulting pellets was checked using DSC (Figure 1). The pure amlodipine had a sharp endothermic melting peak at 199 °C, while hydrochlorothiazide’s endothermic melting peak appeared at 274 °C, indicating that both drugs exhibited a crystalline nature.

Furthermore, in all formulations containing molar ratios (amlodipine besylate/hydrochlorothiazide) of 2:1, 1:1, and 1:2, the melting point peak of amlodipine besylate disappeared, indicating that it had been converted to an amorphous form. Furthermore, the melting point peak of hydrochlorothiazide was also reduced and broadened, indicating a change in its physical form, specifically a decrease in crystallinity or partial amorphization [30,31]. Then the intensities of the melting peaks of all formulations were determined and compared with the pure hydrochlorothiazide, as its melting point peak did not completely disappear [32]. It was found that the integral of the melting peak of pure hydrochlorothiazide decreased from 21.62 to 0.88 and was 1.13 and 1.64 for pellets containing a molar ratio of 2:1, 1:1, and 1:2, which revealed that the increasing concentration of amlodipine besylate had a beneficial effect for minimizing the crystallinity of hydrochlorothiazide due to the high molecular weight and flexible structure of amlodipine besylate, which may damage the crystalline hydrochlorothiazide lattice and initiate amorphization. Additionally, the possibility of the formation of hydrogen bonds increases proportionally with high amlodipine besylate contents because of the increasing number of hydrogen bond acceptor and donor groups (ester groups and amino groups).

### 2.2. X-Ray Powder Diffraction (XRPD)

The XRPD diffractogram showed peaks of the pure amlodipine besylate diffraction at many positions, with a crystallinity index of 88%, while hydrochlorothiazide had multiple peaks, with a crystallinity index of 92.5%.

**Figure 2:**
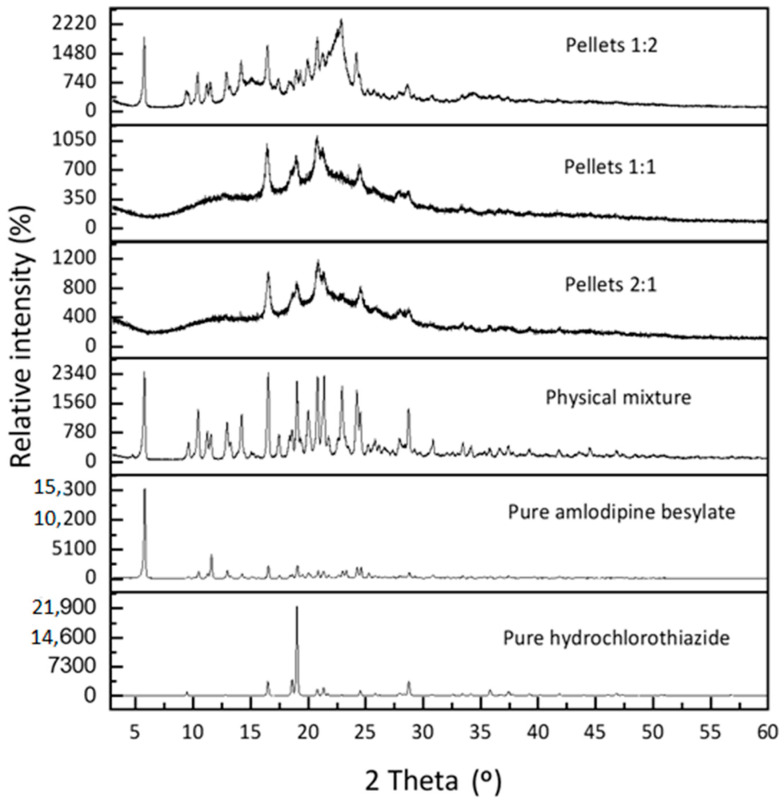
XRPD diffractogram of layered pellets.

According to Figure 2, all those diffraction peaks appeared in the physical mixture, demonstrating the crystalline nature of both drugs, with a total crystallinity index of 90.2% (Table 1).

The diffractogram of all the layered pellet formulations revealed the reduction in all intensities of the diffraction peak and the total net area in addition to the individual net area of each peak [30,31]. The pellets containing a 2:1 molar ratio exhibited the lowest crystallinity index of 26.8%, while the pellets containing a 1:1 and 1:2 molar ratio had a crystallinity index of 33% and 53.6% (Table 1). These results indicated that increasing the concentration of amlodipine besylate in the mixture had a beneficial effect for minimizing crystallinity and enhancing amorphization, and it agreed with DSC results as a result of the decrease in the melting peak intensities of hydrochlorothiazide in different formulations by the same fold (hydrochlorothiazide was incompletely amorphized in the thermogram).

### 2.3. Micro-Computed Tomography (Micro-CT) Measurements

Micro-CT is considered a useful non-destructive tool that can be applied to investigate the internal structure and morphological features of a multiparticulate dosage form, especially layered pellets because it can measure the total and core diameter of pellets [33,34]. On the other hand, it provides segmented images with different colors of different materials that help to distinguish between the pellet core and different layers, in addition to the estimation of the uniformity, thickness, and structural integrity [35].

**Figure 3:**
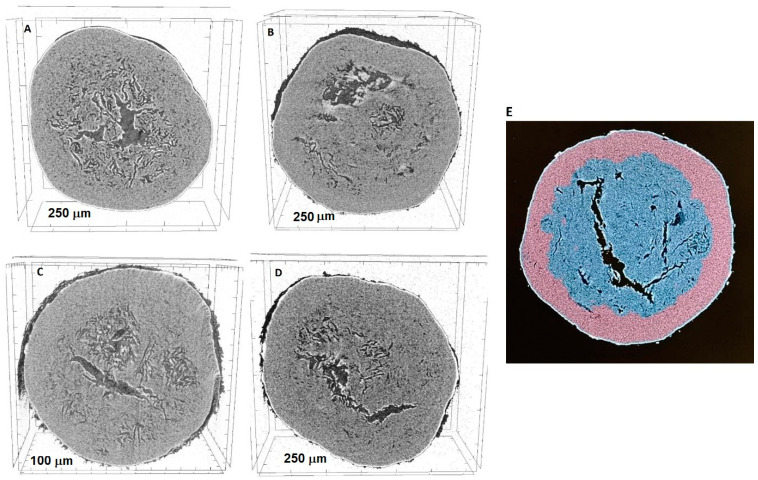
Micro-CT images of (**A**) pure cellet, (**B**) pellet molar ratio 2:1, (**C**) 1:1, (**D**) 1:2, and (**E**) segmented image of pellet molar ratio 1:2.

The examination of the layered pellets and cellets showed the formation of a thin and uniform layer of the drug mixture that completely covered the surface of the pure cellets (Figure 3), and this was confirmed by the increase in the diameter of the pure cellets, as shown in Table 2.

However, the internal part of the resulting pellets contained some cracks and voids that were not related to the application of the mechanical force during the layering process because it also appeared in pure cellets.

### 2.4. Hardness and Mechanical Properties

The breaking hardness of cellets and layered pellets was calculated, and it was found that cellets exhibited a mean breaking force of 19.87 N, while layered pellets exhibited a mean breaking force of 21.6 N, 21.47 N, and 21.82 N for molar ratios of 2:1, 1:1, and 1:2, respectively (Table 2).

Although powder layering was performed without a binder addition, the addition of a powder layer on the pellet surface slightly improved the hardness of the pure cellets. This may be due to the high impeller speed, which increased the movement and mixing between pure cellets and powders. This effect may have led to the production of more contact points and consequently enhanced the adhesion force between the powder and the pellets. Additionally, it increased the filling of the pellet pores and irregularities with the powder, resulting in a higher pellet density and a slight enhancement in hardness. However, there was no significant variation (*p* = 0.198) in the breaking force of the pure cellets and pellets with different molar ratios and the uniformity of the thickness of the layered pellets with different ratios, as mentioned in regard to the results of the micro-CT measurements [36].

**Figure 4:**
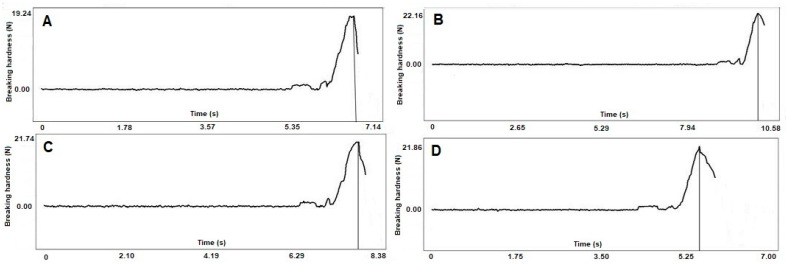
Deformation curves of (**A**) pure cellets; (**B**) pellet molar ratio of 2:1, (**C**) 1:1, and (**D**) 1:2.

It is important to study not only the impact of the breaking hardness but also the deformation curve. It can be seen that the active ingredient layer did not change the deformation process (Figure 4), and no separate breaking point of the layer can be observed.

No differences were observed in the cases of pellets with different ratios of the active ingredient, so this did not affect the deformation process. In all cases, a short elastic phase can be observed at the beginning of the deformation process, followed by a viscoelastic phase and by the cracking of the cellets or pellets, presumably because of the small cavities inside the cellets. This is followed by a relatively long and uniform elastic phase, of which the maximum is the breaking point.

### 2.5. Dissolution Test

The dissolution behavior of the layered pellets was determined as it contributes to the detection of physical changes during the process, as well as the therapeutic efficacy of the drug. Applying a high-shear granulator in the formation of layered pellets is considered a good choice that can improve dissolution due to the decrease in the release time of the drug from pellets (disintegration time) and increased opportunities for contact between the drug mixture on the pellets’ surface and the dissolution medium. Moreover, this process utilized mechanical force, which contributed to changing the physical characteristics of the drugs and promoting amorphization and consequently enhancing the dissolution rate of the drugs. Also, this is a solventless process, which means that it does not require a binder that may retard the drug dissolution.

**Figure 5:**
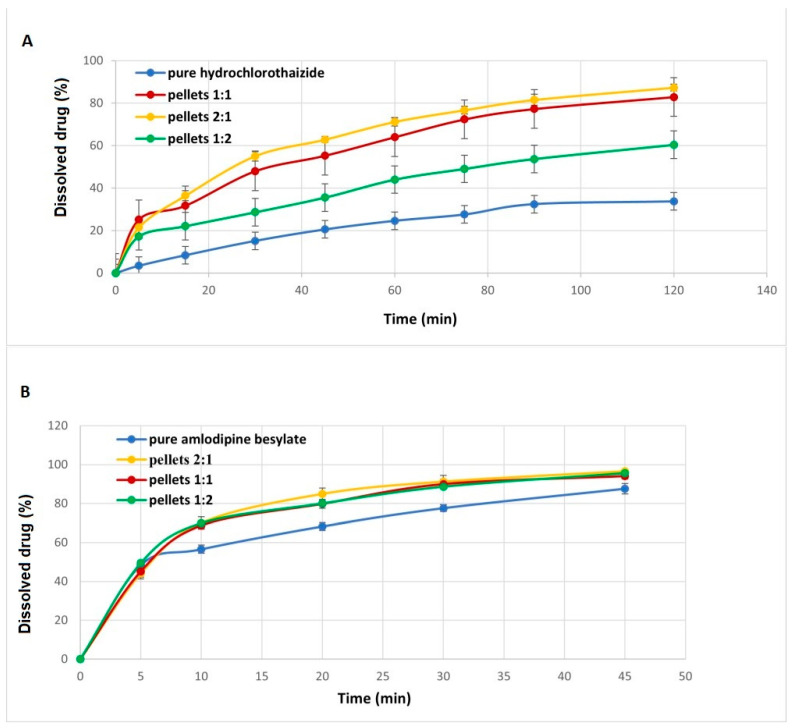
Dissolution release profile for layered pellets containing (**A**) amlodipine besylate and (**B**) hydrochlorothiazide.

Although amlodipine besylate is classified under the Biopharmaceutics Classification System (BCS) as Class I, the dissolution rate was slightly enhanced compared to the crystalline drug by 1.1, 1.07, and 1.09 times for molar ratios of 2:1, 1:1, and 1:2, respectively (Figure 5). Although the dissolution of class 1 is not a rate-limiting step in absorption, the destruction of the crystalline lattice, the formation of supersaturated phases, and the increase in the surface area together were still able to improve the dissolution [37].

However, the dissolution rate of hydrochlorothiazide improved compared with that of the crystalline drug by 2.59, 2.45, and 1.79 times for the molar ratios of 2:1, 1:1, and 1:2, respectively, even though it is not completely converted to an amorphous form. The decrease in the crystallinity index consequently produced a high-energy state in addition to increasing the wettability with the aid of a water-soluble co-former (amlodipine besylate) that contributed to the increase in the dissolution rate of hydrochlorothiazide [3,38].

### 2.6. FTIR

**Figure 6:**
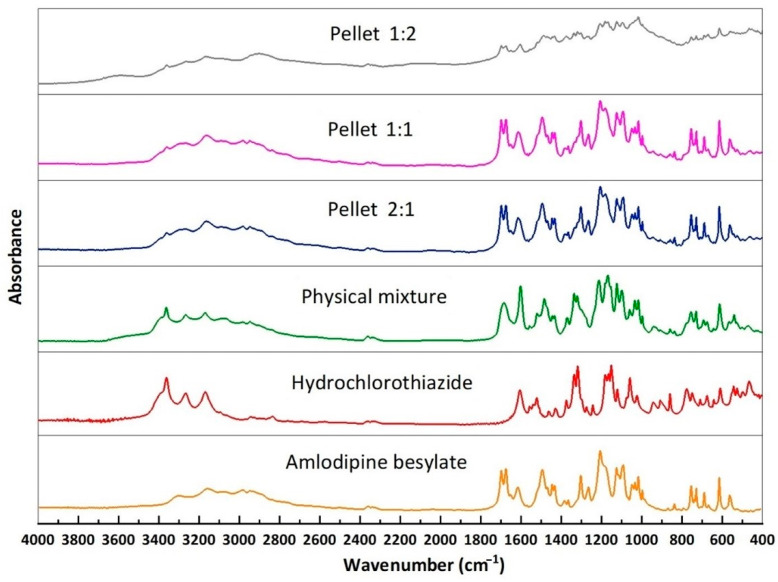
FTIR of pure amlodipine besylate, hydrochlorothiazide, and layered pellets.

FTIR was applied to determine the existence of molecular interactions between hydrochlorothiazide, amlodipine besylate, and cellets. According to Figure 6, the pure amlodipine besylate had characteristic peaks at 3299 cm^−1^ that were related to N–H stretching, specifically of the secondary amine, while the C–H stretching of the aromatic rings appeared at 3159 cm^−1^. The C=C aromatic stretching, C=O stretching of the amide, and the carbonyl group stretching were detected at 1617, 1676, and 1697 cm^−1^, respectively [20].

Pure hydrochlorothiazide exhibited N–H stretching and aromatic C-H stretching at 3361 and 3170 cm^−1^, while asymmetric and symmetric stretching peaks of S=O stretching appeared in a range of 1300–1400 cm^−1^ and 1059 cm^−1^. The aromatic group and the C-N stretching were detected at 1608 and 1244 cm^−1^, respectively [39].

Spectra of all layered pellets with different molar concentrations showed a broadening of the primary sulfonamide and the secondary amine of hydrochlorothiazide, merging with the N-H stretching of amlodipine besylate in a range between 3300 and 3260 cm^−1^ that related to the destruction of the crystalline lattice of the pure drugs [27].

Furthermore, pellets with 2:1 and 1:1 molar ratio spectra exhibited a disappearance of the peak of the symmetric stretching of S=O of hydrochlorothiazide at 1059 cm^−1^, which may be related to the hydrogen bond formation that changed the molecular environment and consequently enforced symmetric stretching and reduced, shifted, or weakened the symmetric peak [40].

Moreover, all other characteristic peaks of the drugs appeared without any shift, and these results indicated that the application of a high-shear granulator influenced the molecular interaction between amlodipine besylate and hydrochlorothiazide.

### 2.7. Stability Studies

**Figure 7:**
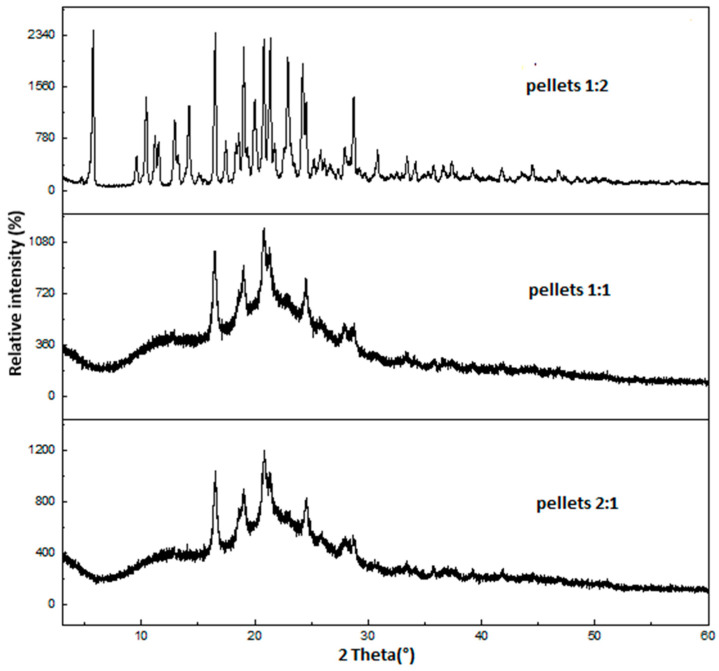
XRPD diffractogram of stability study.

The physical stability of the layered pellets was determined after a storage period of 1 month at 25 °C. The diffractogram of the samples (Figure 7) revealed that the layered pellets remained amorphous at a 2:1 and 1:1 molar ratio throughout storage time due to the appearance of the halo in the XRPD diffractograms, slightly increasing the crystallinity percent by 3 and 1%, respectively, even though both preparations were not completely amorphized.

Furthermore, the 1:2 molar ratio that contained the lowest amount of amlodipine besylate exhibited the highest crystallinity percentage (53.6%) before the stability study and increased to 79.9% after storage and may be related to weak intermolecular interactions that minimized the physical stability of the drug (Table 3).

## 3. Materials and Methods

### 3.1. Chemicals

In this experimental investigation, microcrystalline cellulose spheres with a mean diameter of 1000 μm (Pharmatrans Sanaq AG, Basel, Switzerland) were used as carriers of the drug mixture (cellet), while talc (Molar chemicals Kft, Budapest, Hungary) acted as a glidant. Hydrochlorothiazide and amlodipine besylate were purchased from Sigma-Aldrich (St. Louis, MO, USA). Potassium bromide (J&K Scientific Limited, Beijing, China) was applied in pellet characterization by Fourier transform infrared spectroscopy (FTIR), and the hydrochloric acid solution (0.1 N) was used as the dissolution medium for the resulting pellets.

### 3.2. Preparation of Co-Amorphous Preparation

Hydrochlorothiazide and amlodipine besylate crystals were mixed in various molar ratios using a mortar and pestle; then the drug mixture (6 g) was mixed with MCC spheres (54 g) and talc (0.6 g) in a ProCepT 4M8 granulator (ProCepT NV, Zelzate, Belgium) using an impeller speed of 1500 rpm for 180 min at 60 °C that was retained by a special temperature jacket. Subsequently, the produced pellets were sieved through a 50-mesh screen (Retsch GmbH, Haan, Germany) and stored in a freezer (−20 °C) for further investigation.

### 3.3. Characterization of Layered Pellets

#### 3.3.1. Differential Scanning Calorimetry (DSC)

The thermal properties of the samples were determined using the Mettler-Toledo TGA/DSC1 instrument (Mettler Toledo GmbH, Greifensee, Switzerland). Ten mg of each sample was placed in sealed aluminum pans with 2 holes at the top (100 µL) and inserted into the calorimeter, while the empty pan was used as a reference at a temperature between 25 and 300 °C and a heating rate of 10 °C/min with anhydrous nitrogen at flow rate of 50 mL/min, and then the results were normalized and evaluated using STARe SW 16.30 software.

#### 3.3.2. X-Ray Powder Diffraction (XRPD)

XRPD diffractograms were utilized to determine the transformation in crystal form of all formulations. A total of 10 mg of pure hydrochlorothiazide, amlodipine besylate, and produced pellets were pulverized and tested with a BRUKER D8 advance diffractometer (Bruker AXS GmbH, Karlsruhe, Germany) using Cu Kλ1 radiation (λ = 1.5406) at a voltage of 40 kV and 40 mA, spanning an angular range of 3–60° 2θ at a step time of 0.1 s and a step size of 0.007°. The resulting diffractogram was corrected and analyzed, and then the crystallinity index of all samples was calculated based on DIFFRAC.EVA.V5.2 software.

#### 3.3.3. Micro-Computed Tomography (Micro-CT) Measurements

The morphological and structural characterization of the layered pellets was performed using high-resolution computed tomography (TESCAN UniTOM XL Spectral, TESCAN, Brno, Czech Republic). Samples were scanned using an open-type pumped X-ray source operating at 70 kV tube voltage and 15 W tube power with 3 μm pixel resolution and an exposure time of 255 ms. A total of 2879 projection images were obtained by a 360 ° rotation of the sample with 0.125° rotation step in 39 min scan time. After reconstruction of the images with Panthera (TESCAN, Brno, Czech Republic, version 1.5) software, the volume-rendered 3D CT images were visualized using the same software. AI segmented images of the samples prepared by the “Paint and Segment” method in VGSTUDIO MAX 2023.4 (Volume Graphics).

#### 3.3.4. Hardness and Mechanical Properties

The deformation characteristics and breaking force of the layered pellets and pure cellets were measured by a self-developed texture analyzer that contained a measuring probe working at a speed of 20 mm/min, applying force range of 0–200 N, sensitivity of ±0.5% ±0.1 digit, and with output of 0–5 V. Then the mean and standard deviation of all samples’ breaking force were calculated.

#### 3.3.5. Fourier Transform Infrared Spectroscopy (FTIR)

The first 0.2 g of KBr powder was mixed with different samples and compressed into a disc shape using 10 tons of compression force using a hydraulic press (Specac Inc., Orpington, UK) and then analyzed using Avatar 330 FTIR (Thermo Fisher Scientific Inc., Waltham, MA, USA) at a wavelength range of 400–4000 cm^−1^. Spectra were collected from 128 scans at a spectral resolution of 4 cm^−1^ while applying CO_2_ and H_2_O corrections. The results were normalized and evaluated using Spectragryph 1.2.16.1 software (Friedrich Menges, Obersdorf, Germany).

#### 3.3.6. Dissolution Test

The dissolution of the layered pellets that contained hydrochlorothiazide and amlodipine besylate was carried out in a dissolution apparatus (Ereweka DT 700, Heusenstamm, Germany) using 900 mL of HCl (1.2 N) and a rotation speed of 75 rpm at 37 ± 0.5 °C. Samples were collected at different time intervals (5, 15, 30, 45, 60, 75, 90, and 120 min); then the absorbance of the solutions was measured at λ 238 and 272 nm using UV–Vis spectrophotometer (Unicam Helios α, Spectronic Unicam, Budapest, Hungary).

A calibration curve was prepared by estimating the absorbance of a series of known concentrations of drug solutions (Figure S1) at λ_max_ of 238 nm and 272 nm for amlodipine besylate and hydrochlorothiazide, respectively. Subsequently, the percent of amlodipine besylate release was calculated according to the calibration curve equation (Absorbance = 0.0426 X − 0.0034) with a correlation coefficient R^2^ of 0.9991, while the hydrochlorothiazide calibration curve equation (Absorbance = 0.0139 X − 0.0066) had a correlation coefficient R^2^ of 0.9992.

#### 3.3.7. Stability Study

The layered pellets were placed in a desiccator containing freshly activated silica at 25 °C/5% relative humidity; then the percentage of crystallinity of all samples was determined after 1 month using XRPD.

#### 3.3.8. Statistical Analysis

Statistical analysis, including one-way ANOVA, was used to estimate the presence of significant variation in the breaking hardness between four groups (pure cellets and pellets with a 2:1, 1:1, and 1:2 molar ratio) using Excel software.

## 4. Conclusions

A high-shear granulator was successfully applied to perform the combined layering pelletization and amorphization of the amlodipine besylate and hydrochlorothiazide combination. The DSC and XRPD examination of the resulting pellets confirmed a change in the physical form of both drugs due to the alteration of the endothermic melting peaks and a decrease in the crystallinity percentage of the pellets compared to the physical mixture and the pure drugs by more than 70%. The cumulative release percentage was improved, with good stability, except that the formulation contained a high concentration of hydrochlorothiazide that rapidly recrystallized. However, the micro-CT and texture analyzer results confirmed the success of the layering pelletization, with an increasing pellet thickness and a non-significant change in the pellet hardness compared with MCC pellets. The use of a high-shear granulator is considered an appropriate, novel, and economical technique for obtaining layered pellets containing a drug combination with improved dissolution in a solventless single step, which is also a suitable option for water-sensitive drugs.

## Figures and Tables

**Table 1:** The crystallinity index of the samples.

Samples	Crystallinity Index (%)
pure amlodipine besylate	88.0
pure hydrochlorothiazide	92.5
physical mixture	90.2
1:1 pellet	33.0
2:1 pellet	26.8
1:2 pellet	53.6

**Table 2:** The physical parameters of the samples.

Samples	Diameter (mm)	Breaking Hardness (N)
Pure cellet	1.159 ± 0.042	19.871 ± 2.801
2:1 pellet	1.282 ± 0.017	21.607 ± 3.072
1:1 pellet	1.335 ± 0.013	21.470 ± 3.220
1:2 pellet	1.314 ± 0.074	21.820 ± 3.511

**Table 3:** Crystallinity index of the samples after stability test.

Samples	Crystallinity Index (%) at T_0_	Crystallinity Index (%) After Stability Test
1:1 pellet	33.0	34.0
2:1 pellet	26.8	29.1
1:2 pellet	53.6	79.9

## Data Availability

Data is contained within the article and Supplementary Materials.

## Supplementary Materials

The following supporting information can be downloaded at: https://www.mdpi.com/article/10.3390/ph18101496/s1, Figure S1: Calibration curve of A. hydrochlorothiazide and B. amlodipine besylate; Table S1: Hardness tests; Table S2: Dissolution test and release percent.
