# Supplementary material for: The Development and Characterization of Layered Pellets Containing a Combination of Amorphized Amlodipine Besylate and Hydrochlorothiazide Using a High-Shear Granulator
Source: Pharmaceuticals (Basel). 2025 Oct 5;18(10):1496. doi: 10.3390/ph18101496 (PMC12566888; doi:10.3390/ph18101496)
Supplement: Supplementary file 1 [file pharmaceuticals-18-01496-s001.zip › pharmaceuticals-3844778-supplementary.pdf]

1. Hardness tests

**Numb.**	**Empty cellets**	**Pellets 2:1**	**Pellets 1:1**	**Pellets 1:2**
**1**	20.23	21.3	21.64	25.47
**2**	17.1	24.7	27.67	21.4
**3**	17.04	21.51	17.55	25.03
**4**	17.82	22.75	17.07	16.97
**5**	17.05	24.6	21.99	18.1
**6**	16.1	23.53	25.39	23.13
**7**	18.08	23.86	18.96	24.42
**8**	19.18	25.9	24.86	24.21
**9**	24.46	17.93	27.46	17.08
**10**	22.87	26.39	21.76	22.8
**11**	24.2	21.99	19.98	22.74
**12**	21.34	23.6	18.24	17.16
**13**	23.04	21.94	20.08	16.28
**14**	18.62	16.4	23.79	22.4
**15**	19.27	21.16	21.59	22.19
**16**	20.09	22.95	16.32	24.2
**17**	17.67	16.9	22.94	16.09
**18**	22.9	20.24	22.52	24.9
**19**	23.8	17.19	20.01	26.1
**20**	16.56	17.3	19.58	25.73
**Mean**	19.871	21.607	21.47	21.82
**SD**	2.8	3.07	3.22	3.51

2. Dissolution test and release percent

2.1. Pure hydrochlorothiazide

**Time**	**1**	**2**	**3**	**4**	**5**	**6**	**Mean**	**SD**
0	0	0	0	0	0	0	0	0
5	1.86	4.21	2.05	3.16	4.09	3.79	3.451428571	1.160279113
15	4.92	5.74	5.39	9.51	8.62	9.77	8.421428571	2.212227384
30	11.96	14.57	14.86	16.14	16.1	17.28	15.15166667	1.844780926
45	16.83	21.14	20.37	20.73	21.7	22.47	20.54	1.962630887
60	21.91	23.99	23.61	24.57	25.88	27.55	24.585	1.945700388
75	25.38	25.51	28.95	28.94	30.41	26.16	27.55833333	2.138872756
90	29.74	30.85	30.9	34.68	35.46	32.95	32.43	2.305957502
120	34.49	31.07	34.26	36.22	35.19	31	33.705	2.177840674

2.2. Pure amlodipine besylate

**Time**	**1**	**2**	**3**	**4**	**5**	**6**	**Mean**	**SD**
0	0	0	0	0	0	0	0	0
5	51.19	46.28	47.02	46.1	50.3	49.32	48.36833333	2.187385807
10	58.3	54.96	54.11	55.07	58.29	58.49	56.53666667	2.026066797
20	69.52	66.84	66.19	66.4	70.24	70.46	68.275	2.00537029
30	78.64	75.34	76.13	76.44	80.26	78.89	77.61666667	1.920090276
45	90.21	85.3	85.46	85.51	90.17	89.07	87.62	2.441851756

1.2. Pellets (2:1)

A. hydrochlorothiazide

Time	1	2	3	4	5	6	Mean	SD
0	0	0	0	0	0	0	0	0
5	26.13	19.33	17.85	18.24	23.96	23.28	21.465	3.444083332
15	36.18	37.52	37.45	34.96	32.78	39.62	36.41833333	2.36430469
30	53.13	55.94	55.3	54.59	52.42	58.99	55.06166667	2.332049885
45	60.49	63.19	63.92	63.52	61.83	64.1	62.84166667	1.40580819
60	69.25	71.13	72.36	72.09	68.45	73.82	71.18333333	2.018431734
75	75.43	77.28	76.11	77.42	74.32	79.56	76.68666667	1.82530728
90	77.82	82.8	82.9	82.9	78.65	84.1	81.52833333	2.60883435
120	85.76	88.3	87.64	87.95	84.99	89.32	87.32666667	1.632294908

B. amlodipine besylate

Time	1	2	3	4	5	6	Mean	SD
0	0	0	0	0	0	0	0	0
5	45.92	46.19	41.23	47.62	41.83	41.8	44.09833333	2.783906727
10	72.38	72.24	66.82	73.88	67.13	67.63	70.01333333	3.152825188
20	87.69	87.86	81.5	87.44	81.75	83.09	84.88833333	3.090471916
30	93.55	93.75	86.92	94.61	88.1	90.43	91.22666667	3.230174402
45	97.96	97.47	94.95	96.89	96.42	95.56	96.54166667	1.140761442

2.1. Pellets (1:1)

A. hydrochlorothiazide

Time	1	2	3	4	5	6	Mean	SD
0	0	0	0	0	0	0	0	0
5	26.49	24.38	24.84	23.72	23	28.99	25.23666667	2.184148957
15	33.41	30.15	31.66	32.58	29.49	33.1	31.73166667	1.60876868
30	51.33	46.85	46.27	49.13	47.71	46.55	47.97333333	1.942345661
45	57.42	54.2	50.84	56.86	54.74	57.49	55.25833333	2.574058404
60	64.11	62.47	62.51	63.23	66.05	65.52	63.98166667	1.528193923
75	72.49	71.63	71.2	73.82	71.53	73.47	72.35666667	1.090681744
90	76.82	75.34	75.49	78.13	78.2	79.52	77.25	1.65893942
120	82.95	81.57	80.96	84.01	84.26	83.54	82.88166667	1.34344954

B. amlodipine besylate

Time	1	2	3	4	5	6	Mean	SD
0	0	0	0	0	0	0	0	0
5	41.77	46.26	41.98	44.37	47.8	48.92	45.18333333	2.984712158
10	66.33	69.22	67.81	68.14	70.03	70.98	68.75166667	1.669987026
20	77.91	79.86	77.48	78.62	82.76	82.35	79.83	2.26314825
30	86.87	90.94	87.41	92.13	92.82	89.77	89.99	2.44663851
45	91.92	94.83	92.73	94.97	94.3	95.84	94.09833333	1.482341616

3.1. Pellets (1:2)

A. hydrochlorothiazide

Time	1	2	3	4	5	6	Mean	SD
0	0	0	0	0	0	0	0	0
5	14.94	15.71	19.02	18.73	16.94	18.09	17.23833333	1.663242817
15	20.98	19.86	25.89	21.86	19.76	24.08	22.07166667	2.452952643
30	26.35	25.48	31.25	30.2	26.31	32.17	28.62666667	2.910812029
45	33.48	33	37.55	36.45	33.95	38.81	35.54	2.399349912
60	41.16	40.51	45.01	48.22	41.96	47.13	43.99833333	3.256767825
75	46.72	47.23	49	52.34	47.11	51.9	49.05	2.508146726
90	50.88	51.62	56.39	55.85	51.74	55.67	53.69166667	2.524261608
120	58.4	58.96	62.71	62.79	56.89	62.58	60.38833333	2.615090183

B. amlodipine besylate

Time	1	2	3	4	5	6	Mean	SD
0	0	0	0	0	0	0	0	0
5	48.71	53.69	48.02	51.62	49.82	44.75	49.435	3.082354619
10	67.95	72.66	68.21	72.89	70.05	67.49	69.875	2.410109956
20	78.32	81.61	78.44	82.7	81.39	77.54	80	2.150432515
30	85.16	90.48	86.37	92.63	92.11	84.87	88.60333333	3.54442473
45	94.89	96.89	94.97	95.82	96.63	94.02	95.53666667	1.108759066

Calibration curves

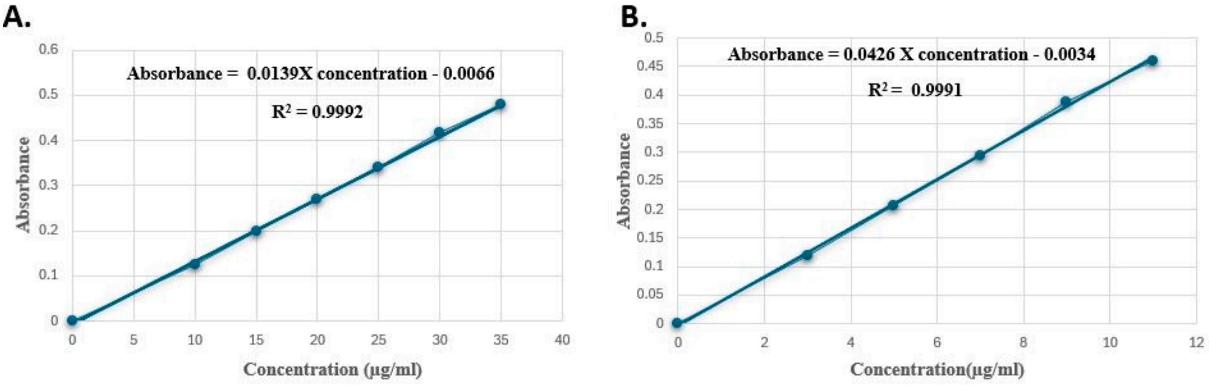

Figure S1. Calibration curve of A. hydrochlorothiazide and B. amlodipine besylate
